# New Species of Soldier Fly—*Sargus bipunctatus* (Scopoli, 1763) (Diptera: Stratiomyidae), Recorded from a Human Corpse in Europe—A Case Report

**DOI:** 10.3390/insects12040302

**Published:** 2021-03-30

**Authors:** Marek Michalski, Piotr Gadawski, Joanna Klemm, Krzysztof Szpila

**Affiliations:** 1 Department of Experimental Zoology and Evolutionary Biology, Faculty of Biology and Environmental Protection, University of Lodz, Banacha Street 12/16, 90-237 Łódź, Poland; 2 Department of Invertebrate Zoology and Hydrobiology, Faculty of Biology and Environmental Protection, University of Lodz, Banacha Street 12/16, 90-237 Łódź, Poland; piotr.gadawski@biol.uni.lodz.pl; 3 Facility of Forensic Medicine Barzdo i Żydek, Franciszkańska Street 104/112, 91-845 Łódź, Poland; joanna.monika.klemm@gmail.com; 4 Department of Ecology and Biogeography, Faculty of Biological and Veterinary Sciences, Nicolaus Copernicus University, Lwowska Street 1, 87-100 Toruń, Poland; szpila@umk.pl

**Keywords:** carrion, larva, first record, barcoding DNA, integrative taxonomy

## Abstract

**Simple Summary:**

In the current study, we present the first record of twin-spot centurion fly larvae, *Sargus bipunctatus* (Scopoli, 1763), feeding on a human corpse. The morphology of collected imagines and larvae of *S. bipunctatus* was documented, and a standard COI barcode sequence was obtained. Morphology- and DNA-based methods were used to distinguish the larvae of *S. bipunctatus* and its relative, *Hermetia illucens* (Linnaeus, 1758). The potential of *S. bipunctatus* for practical applications in forensic entomology is currently difficult to assess.

**Abstract:**

The only European Stratiomyidae species known for feeding on human corpses was the black soldier fly *Hermetia illucens* (Linnaeus, 1758). Analysis of fauna found on a human corpse, discovered in central Poland, revealed the presence of feeding larvae of another species from this family: the twin-spot centurion fly *Sargus bipunctatus* (Scopoli, 1763). The investigated corpse was in a stage of advanced decomposition. The larvae were mainly observed in the adipocere formed on the back and lower limbs of the corpse, and in the mixture of litter and lumps of adipocere located under the corpse. Adult specimens and larvae were identified based on morphological characters, and final identification was confirmed using DNA barcoding. Implementing a combination of morphological and molecular methods provided a reliable way for distinguishing the larvae of *S. bipunctatus* and *H. illucens*. The potential of *S. bipunctatus* for practical applications in forensic entomology is currently difficult to assess. Wide and reliable use of *S. bipunctatus* in the practice of forensic entomology requires further studies of the bionomy of this fly.

## 1. Introduction

The family Stratiomyidae (soldier flies), representing the suborder of Brachycera Orthorrhapha, includes more than 2600 described species [1]. Large stratiomyiids are often characterized by their mimicry of wasps or bees (Hymenoptera: Aculeata). Larvae of these flies are dorso-ventrally flattened, with strongly sclerotized integument. Their cuticle has a polygonal pattern due to numerous calcareous incrustations. The larvae of the majority of species live in terrestrial, humid environments, with the exception of a few typically aquatic species. Terrestrial species usually feed on dead organic matter, e.g., humus, decaying parts of the plants and fungi, and the faeces of vertebrates and invertebrates. Stratiomyidae utilize a mode of pupation unique among the Orthorrhapha, pupating inside the cuticle of the last larval instar [2,3].

The only species of Stratiomyidae with confirmed forensic importance is the black soldier fly *Hermetia illucens* (Linnaeus, 1758). This species, probably native to Central America, can currently be found in warmer regions around the world. In the northern part of North America, it reaches the province of Ontario [4]. In Central Europe, it is distributed from the south up to the Czech Republic [5]. The larvae of *H. illucens* are polyphagous, being able to feed on almost any type of decaying plant or animal matter. Since the 1970s, these larvae have been often used to accelerate the decomposition of organic waste, and bred as food for poultry, pigs, fish, terrarium animals, or as fishing baits [6]. As a result, the species is continuously transferred to new territories [4]. Larvae of black soldier fly have been known to be forensically important since 1915 [7]. They feed on carcasses in a phase of advanced decomposition, and are active only at temperatures exceeding 20 °C. At an optimal temperature of 30 °C, the full development cycle from hatch to maturity takes 43 days [8]. Larvae are also used to estimate the postmortem interval (PMI) in cases where several weeks have passed since death [9]. However, the use of *H. illucens* larvae to determine PMI based on the development approach is quite problematic, due to their presence on highly decomposed corpses, when the time between death and oviposition is long and difficult to estimate [9]. Moreover, specimens from different populations of this species may differ significantly in the rate of development, so that the broad use of developmental models established for particular populations is not valid [8].

Entomological material collected from a recent case from Poland indicates that *H. illucens* is not the only species of Stratiomyidae that can successfully develop on human corpses. Stratiomyiid larvae were collected feeding on human remains and identified, using DNA barcoding and morphological characters, to be *Sargus bipunctatus* (Scopoli, 1763). The trophic relationship of *S. bipunctatus* with dead organic matter of animal origin has already been mentioned by Chick [10]. However, this study marks the first record of larvae of this species feeding on human remains, thereby extending the list of European fly species potentially important for medico–legal purposes.

## 2. Case Description

**Figure 1:**
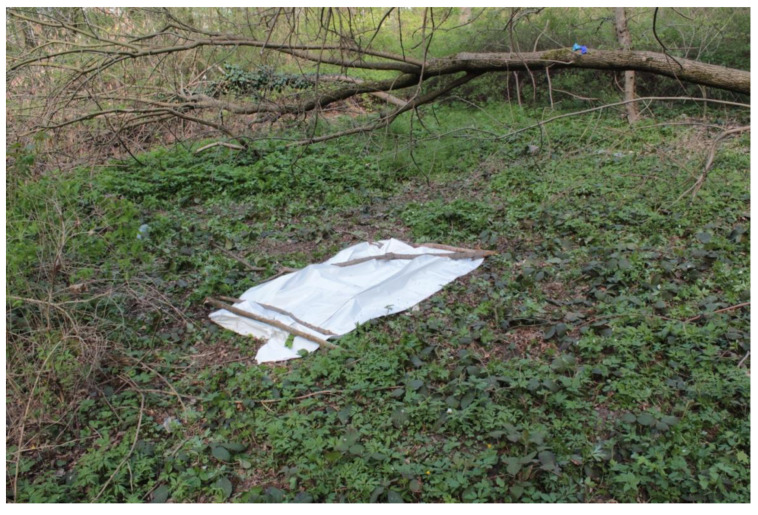
The site of the corpse disclosure, 22 April 2019. Photo—M. Michalski.

An unidentified human corpse in an advanced stage of decomposition was found in the City of Lodz (central Poland) on the evening of 21 April 2019, in Jozef Pilsudski park (51°776278′ N, 19°400248′ E). It was located in a small clearing surrounded by dense vegetation, 10 m from a small open river channel. The body was lying in an anatomical position on the ground covered with creeping vegetation, mainly blackberry (*Rubus* L.) (See Figure 1).

The corpse was dressed in an undershirt and long denim trousers. The head and torso were almost completely skeletonized. Soft tissues of the upper back and the proximal parts of the upper limbs were mummified. The lower parts of the back and the tissues of the lower limbs, covered by denim trousers, had changed to adipocere. Gnaw marks on the feet phalanges and the presence of faeces indicated the activity of vertebrate scavengers. Based on the morphological features of the skeleton examined by the forensic physician, it was initially estimated that the human remains belonged to a woman aged 25–45 years. During the examination, as well as during the subsequent autopsy, no antemortem injuries were found. Therefore, it was impossible to establish the circumstances and cause of death. 

After the body examination, the remains were taken to the morgue. The entomological material was collected at the site of their disclosure the next morning. The supplementary material was collected during the autopsy and subsequent body examination on the 23 April 2019. The collected material was preserved in 75% ethyl alcohol. 

Imagines of predatory beetles, from the families Staphylinidae and Histeridae, were most dominant on the corpse. A few specimens of *Omosita* spp. (Nitidulidae), *Necrobia violacea* Linnaeus, 1758 (Cleridae), *Thanatophilus sinuatus* (Fabricius, 1775), and *Oiceoptoma thoracicum* (Linnaeus, 1758) (Silphidae) were also found. Among the flies, the most abundant were larvae of the Piophilidae family feeding in the adipocere. Numerous larvae and pupae of *Fannia* sp. were collected from the folds of the clothes, and a few Muscid larvae belonging to the genera *Hydrotaea* Robineau-Desvoidy, 1830 and *Muscina* Robineau-Desvoidy, 1830 were collected from the soft tissue residue. Three empty puparia of *Chrysomya albiceps* (Wiedemann, 1819), attached to clothing, were the only indicators of the presence of blow flies. 

Several larvae, ~1 cm long, were collected from the folds of the clothes covered with litter and adipocere formed on the back and lower limbs of the corpse. The specimens, covered with moist soil mixed with organic matter, were preliminarily identified as larvae of *H. illucens*. After cleaning in an ultrasonic cleaner, the surface of the larvae was re-examined and showed a clear striped pattern, uncharacteristic for *H. illucens*. 

**Figure 2:**
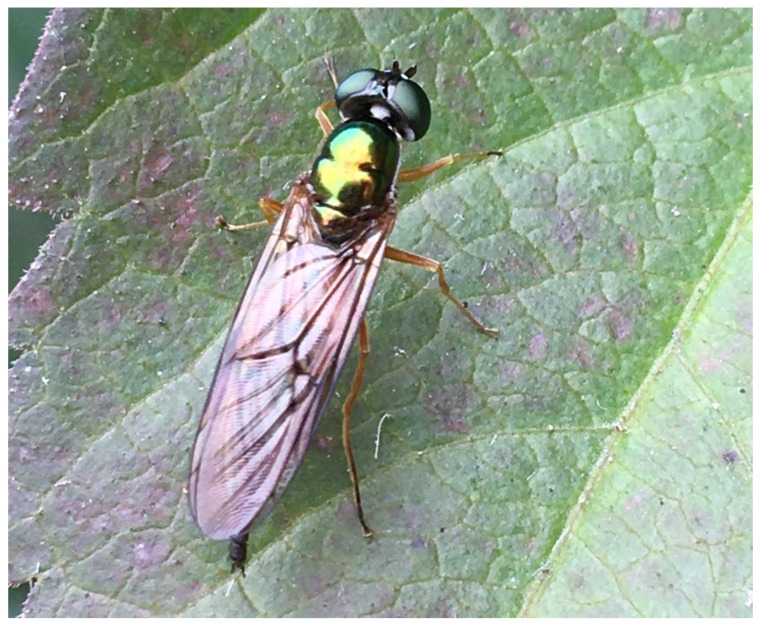
Imago of *Sargus bipunctatus*. Łódź—Zoo, 1 October 2019. Photo—M. Michalski.

On the 1 October 2019, numerous mature specimens of *Sargus bipunctatus* were found in the compost and manure dumping place in the Łódź Zoo, located approximately 1.1 km away from the site where the body was discovered (Figure 2). This is likely the primary origin of *S. bipunctatus* specimens in the area.

## 3. Material and Methods

Larval specimens were identified first as the genus *Sargus* Fabricius, 1798 [=*Geosargus* Bezzi, 1907] based on available literature [11], then to the species level based on the keys provided by Rozkošny [12]. Species-level identification and subsequent photographic documentation was performed using a Leica M205 FA stereo microscope (Leica Microsystems GmbH, Wetzlar, Germany) with imaging software provided by the manufacturer. FOCUS Projects 4 Professional software (Franzis Verlag GmbH, Haar, Germany) was used to perform photo stacking. To confirm species identifications, the barcode region of *cytochrome oxidase unit I* (COI) was amplified from DNA extracted from three specimens, and compared to data stored in online repositories. DNA extraction was performed in the Molecular Laboratory of the Department of Invertebrate Zoology and Hydrobiology at the University of Lodz, Poland. DNA was extracted from tissue dissected from the anal segments of the larvae using a GeneMATRIX Tissue DNA Purification Kit (EURx, Gdansk, Poland), following manufacturer protocol. Dissected tissue was incubated overnight in lysis buffer with Proteinase K. The 658 bp barcode region of COI was then amplified for each specimen using a Polymerase Chain Reaction (PCR) and the standard barcode primer pair, LCO1490/HCO2198 (Biomers.net GmbH, Ulm, Germany, [13]). A PCR was performed in a final volume of 11 µL reaction mix, containing 5 µL of DreamTaq reaction Buffer (ThermoFisher Scientific, Waltham, MA, USA), 0.8 µL of LCO1490 primer, 0.8 µL of HCO2198 primer, 2.4 µL of ultrapure water, and 2 µL of DNA template. The PCR conditions consisted of 94 °C for 1 min followed by 5 cycles of 30 s at 94 °C, 1 min 30 s at 45 °C and 1 min at 72 °C; 36 cycles at 94 °C for 30 s, 51 °C for 1 min 30 s, and 72 °C for 1 min; with the final extension of 5 min at 72 °C. Amplification success was confirmed via visualisation using agarose gel electrophoresis. PCR products were purified using a mix of FastAP (1 U/µL, ThermoFisher Scientific, Waltham, MA, USA) and Exonuclease I (20 U/µL ThermoFisher Scientific, Waltham, MA, USA). Direct sequencing of the PCR product with the marker-specific primers was outsourced to Macrogen Europe (Amsterdam, The Netherlands). The obtained COI sequences were edited and primers removed using Geneious Pro 11 (Biomatters Ltd., Auckland, New Zealand [14]). From three analysed specimens, only one DNA extraction was successful, and provided a good quality sequence (617 bp). The identity of the obtained COI sequence was verified using the Barcode of Life Data System (BOLD) Identification Engine [15]. As a result, a list of twenty top matches was obtained with 99.82% similarity, with all sequences belonging to *Sargus bipunctatus* (Scopoli, 1763) (BIN URI: BOLD:ACI9008). Nineteen records were collected in Vancouver, Canada, and one from Frankfurt, Germany. The obtained COI sequence was then deposited in the BOLD v4 and GenBank online repositories under accession numbers: BOLD Process ID: DPTPL001-21 (Sample ID: DptPL_Lodz_LA_1); GenBank: MW661345 (dx.doi.org/10.5883/DS-DIPTPL) to make it available for future studies [15,16].

## 4. Discussion

The twin-spot centurion fly, *Sargus bipunctatus* (Scopoli, 1763) [=*Chrysochroma bipunctatum* (Scopoli, 1763); *Sargus bipunctatus* Costa, 1844], is widespread in Europe and the northwest of North America [1,11,17]. Single records are also known from the mountainous areas in Iran, Tunisia, and Turkey [1,18,19,20]. According to Nartshuk [21], it is a Euro–Caucasian species associated with temperate deciduous forests. The larvae of this fly feed in terrestrial environments on various substrates, such as decaying plant debris, compost, and the faeces of vertebrates [11,22,23,24]. According to Dušek and Rozkošny [25], larvae can also be found in egg sacs of the Moroccan locust *Dociostaurus moroccanus* (Thunberg, 1815), and Oldroyd [26] recorded larvae in decaying mushrooms of the species, *Cerioporus squamosus* (Huds.) Quélet (1886). The variety of substrates from which larvae of *S. bipunctatus* has been collected indicates a broad feeding spectrum. Despite this, only Chick [10] has reported the relationship between larvae of *S. bipunctatus* and decaying animal remains. Numerous females of the species were observed on the carcass of a domestic pig on the 24th day of decomposition. Nineteen days later, larvae were found feeding in a mixture of soil, mulch, and the putrefactive liquid exuding from the carcass. Sukontason et al. [27] recorded single cases of *Sargus* sp. larvae feeding on human bodies found in the forests of Thailand from 2000–2006.

**Figure 3:**
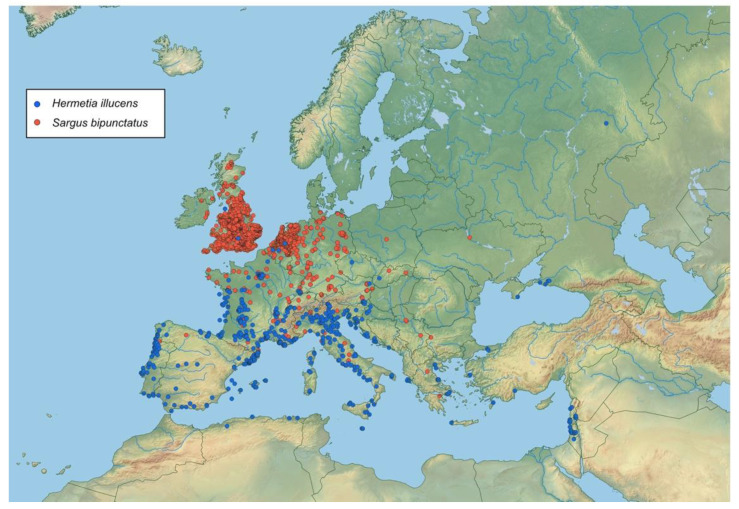
Distribution of *Hermetia illucens* and *Sargus bipunctatus* in Western Palearctic, based on data coming from GBIF and iNaturalist.

**Figure 4:**
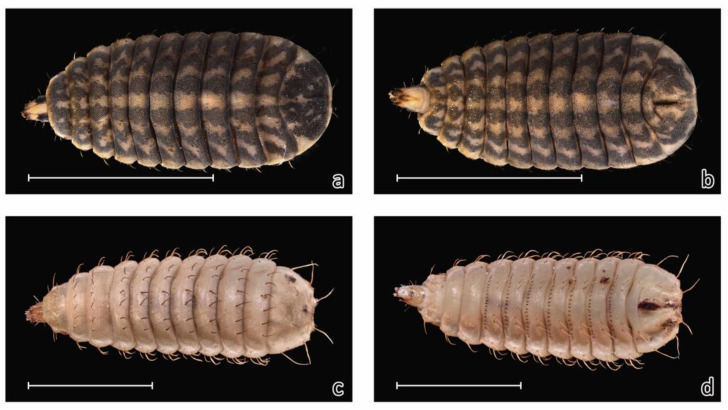
Third instar larvae of *Sargus bipunctatus*. (**a**): upperside, (**b**): underside and *Hermetia illucens*, (**c**): upperside, (**d**): underside. Scale bar: 5 mm. Photos—M. Michalski.

Larvae of *S. bipunctatus* are very distinctive and easy to distinguish from the specimens of other necrophagous flies by their dorsoventral flattening. The only species possible to misidentify it with is *Hermetia illucens*, which belongs to the same family Stratiomyidae. The geographic distributions of both species overlap [17,28,29,30] (see Figure 3). Moreover, it is expected that the northern range of *H. illucens* will expand through both natural processes and the unintentional release of mature flies from black soldier fly breeding farms. Larvae of *S. bipunctatus* and *H. illucens* can be easily distinguished based on morphological features alone. *Sargus bipunctatus* has a distinct colouration pattern, with six longitudinal stripes on its abdominal segments, and very short dorsal, dorsolateral, lateral and ventral bristles (Figure 4).

Due to the characteristic morphology of both larvae (Figure 4) and adult forms (Figure 2), this species is difficult to overlook when observing its food substrate. Despite this, larvae of this species have not been recorded on the corpses of large vertebrates in any succession experiments conducted in Central Europe to date [31,32,33,34,35,36]. As such, the presence of *S. bipunctatus* in this case, may only be an incidental colonization of human remains. At the current time, it is difficult to evaluate the potential of this species in forensic applications, due to the narrow range of environments included in the studies of insect succession on carcasses in Central Europe [31,32,33]. In the discussed case, there is a significant correlation between the presence of larvae and the stage of advanced decomposition. This relation indicates that the species could be used for the estimation of postmortem interval, using a method based on successional patterns rather than development rate.

Imagines of *S. bipunctatus* are active from July to November, with maximum abundances recorded from September to October [28]. In the analysed case, the presence of fully developed larvae of *S. bipunctatus* in spring may indicate that the corpse was in the advanced decomposition stage during the period of imagines activity, probably in the fall of the previous year. This hypothesis may also be confirmed by the observed coexistence of fully grown larvae of Muscidae and empty puparia of *Chrysomya albiceps* [35]. More detailed conclusions are not currently possible due to the lack of precise data on the development of *S. bipunctatus*. Access to such information is a crucial issue for the practical use of forensic entomology [37]. Based on the well-planned field and laboratory experiments, even relatively rare insects may be considered highly important for medico–legal purposes. A good example is the beetle, *Necrodes littoralis* (Linnaeus, 1758), included in the red lists of threatened animals in Central European countries [38,39]. Analysis of specimens collected in real cases and during insect succession studies has shown its frequent presence on large vertebrate carrion, including human corpses [33,40]. Such knowledge stimulated extensive studies of *N. littoralis* development, conducted under laboratory conditions [41,42]. Finally, the presence of immature stages of this beetle was used for the estimation of the time of death [43,44]. We hope that this model path from the laboratory studies to casework will be, at least partly, successfully replicated for *S. bipunctatus.* Further studies of this species should have a precise forensic profile and cover field studies of environmental preferences, preferred food sources and habitats, role in necrophagous insect community, and activity period during successional changes of carrion, as well as laboratory experiments concerning thermal requirements during the development of immature life stages.

## Data Availability

The obtained COI sequence is available at: Barcode of Life Data System (BOLD) (http://boldsystems.org), Process ID: DPTPL001-21 (Sample ID: DptPL_Lodz_LA_1). GenBank: (https://www.ncbi.nlm.nih.gov/genbank/) Genbank Accession number: MW661345 (dx.doi.org/10.5883/DS-DIPTPL).
